# CDC consultations related to ophthalmologic practices and settings, 2016–2021

**DOI:** 10.1017/ash.2023.402

**Published:** 2023-09-29

**Authors:** Kevin Spicer, Joseph Perz, Kiran Perkins

## Abstract

**Background:** Healthcare activities that include instrumentation or manipulation of mucosal tissue or normally sterile sites, such as the eye and its associated structures, place patients at risk of infectious and other complications. We reviewed queries to the CDC Prevention and Response Branch that were focused on ophthalmologic procedures and settings to examine opportunities to improve infection prevention and control (IPC) practices in these settings. **Methods:** We reviewed internal CDC consultation records received from January 1, 2016, through December 31, 2021, to identify those involving ophthalmologic procedures or settings. Consultations were reviewed to determine setting type, number of patients affected, organisms identified, nature of infection control breaches, and whether medical products were implicated. Descriptive statistics were calculated. **Results:** We identified 24 consultations among 19 states and US territories. Of these, 21 (87.5%) involved outpatient settings, of which 9 (43%) were ambulatory surgery centers. Consultations included the following non–mutually exclusive categories. There were 18 adverse postsurgical events (75%), such as mycobacterial infection after laser surgery and toxic anterior segment syndrome following cataract surgery (n = 5). There were 11 infections following ophthalmologic clinical care (46%), such as epidemic keratoconjunctivitis due to adenovirus 8. There were 8 suspected medication-related events (33%) including contamination of ophthalmic medication when manufactured or compounded offsite. There were 8 medical-device reprocessing concerns (33%) including inappropriate high-level disinfection. There were 8 instances of improper environmental cleaning and disinfection (33%), for example, during an adenovirus outbreak. There were 3 cases of potential mishandling of medications onsite (12.5%), such as multiuse eye drops. Also, 3 events (12.5%) were associated with potentially contaminated donor tissue, such as corneas for transplantation. When a consultation included identification of a pathogen (n = 11), organisms included bacteria (n = 7, 64%), viruses (n = 2, 18%), and fungi (n = 3, 27%). In total, 202 patients had confirmed ophthalmologic infections or adverse events. **Conclusions:** Based on our review of recent outbreaks, healthcare personnel in ophthalmologic settings may have deficits in training related to instrument reprocessing and environmental cleaning specific to ophthalmic equipment and settings that can result in harm to patients. These settings could benefit from targeted training to improve IPC practices specific to ophthalmologic examinations and procedures. This review was limited to analysis of investigations that were voluntarily reported to the CDC. A formal surveillance system for adverse outcomes in this setting could clarify the nature and frequency of IPC issues of greatest concern.

**Disclosures:** None

